# Isolated Tuberculous Testicular Abscess Without Systemic Involvement: A Rare Cause of Scrotal Swelling

**DOI:** 10.31486/toj.26.0012

**Published:** 2026

**Authors:** Bharti Varshney, Sunita Agarwal, Yogi Raj Joshi

**Affiliations:** Department of Pathology, Dr. Sampurnanand Medical College, Jodhpur, Rajasthan, India

**Keywords:** *Abscess*, *orchitis*, *testis*, *tuberculosis*

## Abstract

**Background:**

Genitourinary tuberculosis most commonly involves the epididymis; isolated testicular involvement is rare and usually presents as a mass mimicking malignancy. Genitourinary tuberculosis presenting as a localized testicular abscess without evidence of tuberculosis elsewhere in the body is particularly uncommon.

**Case Report:**

A 60-year-old male presented with right scrotal swelling of 2 months’ duration associated with low-grade fever. He reported no urinary symptoms and no weight loss. Clinical examination revealed an enlarged right testis with loss of normal testicular architecture. Ultrasonography showed a heterogeneous hypoechoic lesion predominantly replacing the right testis, with central liquefaction suggestive of an intratesticular abscess. The contralateral testis was normal. Routine blood tests, renal and liver function, and tumor markers (alpha-fetoprotein, beta-human chorionic gonadotropin, lactate dehydrogenase) were within normal limits. Chest radiograph and abdominal ultrasound excluded pulmonary and renal tuberculosis. The patient underwent right inguinal orchidectomy for diagnosis and management. Histopathology demonstrated granulomatous orchitis with caseous necrosis and Langhans giant cells consistent with a tubercular etiology. No evidence of malignancy was seen. Postoperatively, the patient received a 6-month course of first-line antitubercular therapy. The patient had a good clinical recovery, and at follow-up had no evidence of disease.

**Conclusion:**

In endemic regions, isolated tuberculous testicular abscess should be considered in the differential diagnosis of testicular abscesses. Orchidectomy is useful diagnostically and therapeutically for patients with irreversibly damaged testicular tissue or suspicion of malignancy.

## INTRODUCTION

Tuberculosis is a major global health burden, and extrapulmonary tuberculosis accounts for approximately 10% of cases.^1^ Genitourinary tuberculosis, the second most common form of extrapulmonary tuberculosis, accounts for 30% to 40% of extrapulmonary tuberculosis cases, and the epididymis, kidneys, prostate, and seminal vesicles are the most frequently affected sites.^1,2^ Isolated involvement of the testis is rare, as tuberculosis typically spreads from the epididymis through contiguous extension.^3^

Cases of testicular tuberculosis typically present as a solid mass indistinguishable from malignancy, and the condition is often diagnosed only after orchidectomy.^4-6^ Presentation as an isolated intratesticular abscess without clinical, radiologic, or microbiologic evidence of tuberculosis elsewhere is rare.^7,8^ Further, an isolated intratesticular abscess without pulmonary, renal, or epididymal involvement is also uncommon, with only sporadic reports in the literature.^9,10^

We present a case of isolated tuberculous testicular abscess and highlight the diagnostic considerations.

## CASE REPORT

A 60-year-old male (a resident of Jodhpur, Rajasthan, India) presented to the urology clinic with complaints of right scrotal swelling of 2 months’ duration that was associated with a dull aching/dragging pain and intermittent low-grade fever. The patient denied dysuria, urinary frequency, urethral discharge, trauma, anorexia, and unexplained weight loss. He had no history of tuberculosis, no known contact with a tuberculosis patient, and no prior genitourinary instrumentation or high-risk sexual behavior.

On examination, the patient was afebrile and hemodynamically stable. General physical examination did not reveal lymphadenopathy or organomegaly. Abdominal examination was unremarkable, without flank tenderness or suprapubic fullness. Scrotal examination showed an enlarged right hemiscrotum with stretched, mildly erythematous skin. The involved testis was enlarged, firm to fluctuant, and tender with loss of normal testicular contour. The contralateral testis and epididymis were normal to palpation. No inguinal lymphadenopathy or sinus formation was detected.

Laboratory investigations (Table) revealed hemoglobin of 12 g/dL, total leukocyte count of 13,000 cells/μL with mild neutrophilia, and normal renal function tests. Erythrocyte sedimentation rate (48 mm/h) and C-reactive protein (12.6 mg/L) were elevated. Urinalysis was unremarkable without pyuria or hematuria, and urine culture showed no growth. Serology for human immunodeficiency virus was negative. Serum tumor markers, including alpha-fetoprotein, beta-human chorionic gonadotropin, and lactate dehydrogenase, were within normal limits.

**Table.** Hematologic, Biochemical, and Microbiologic Assessment

Parameter		Patient Value	Reference Range
Hemoglobin, g/dL		12	13-17 (males)
Total leukocyte count, cells/μL		13,000	4,000-11,000
Neutrophils, %		79	40-75
Urea, mg/dL		20	6-24
Creatinine, mg/dL		0.9	0.7-1.3
Erythrocyte sedimentation rate, mm/h		48	0-15 (males)
C-reactive protein, mg/L		12.6	<10
Urinalysis	Pus cells, HPF	0-1	0-5
	Blood, HPF	Nil	0-3
Urine culture		No growth	Negative
Human immunodeficiency virus serology		Negative	Negative
Alpha-fetoprotein, ng/mL		4.2	<10
Beta-human chorionic gonadotropin, mIU/mL		1.1	<5
Lactate dehydrogenase, U/L		156	140-280

HPF, high-power field.

Scrotal ultrasonography demonstrated an enlarged right testis predominantly replaced by a heterogeneous hypoechoic lesion with central liquefaction and internal echoes consistent with an intratesticular abscess, along with thickening of the tunica albuginea. Color Doppler showed peripheral hypervascularity with relatively avascular liquefied areas. The epididymis appeared slightly enlarged without discrete nodules. The contralateral testis and epididymis were normal. No hydrocele, scrotal sinus, or calcifications were noted. Chest radiograph was normal, and ultrasound of the kidneys and urinary tract showed no evidence of renal or ureteric lesions suggestive of genitourinary tuberculosis.

Because of the localized intratesticular abscess with loss of normal testicular architecture, diagnostic uncertainty regarding the underlying tumor, and the risk of persistent infection, inguinal orchidectomy was performed. Through a standard inguinal approach, the cord was ligated proximally and the testis delivered. The testis was enlarged with a tense, firm fibrous capsule (tunica albuginea). The specimen was sent for histopathology.

**Figure 1.**
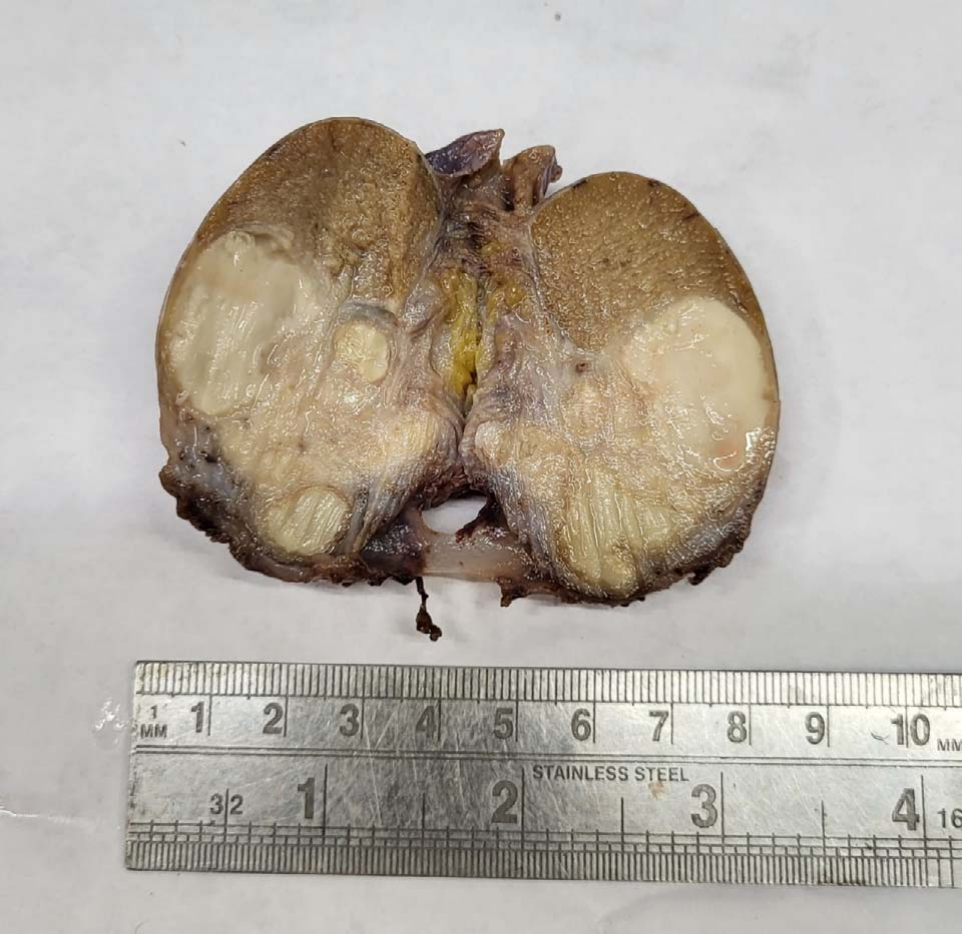
Gross examination of testis shows grey-white nodules and part of the testicular parenchyma.

**Figure 2.**
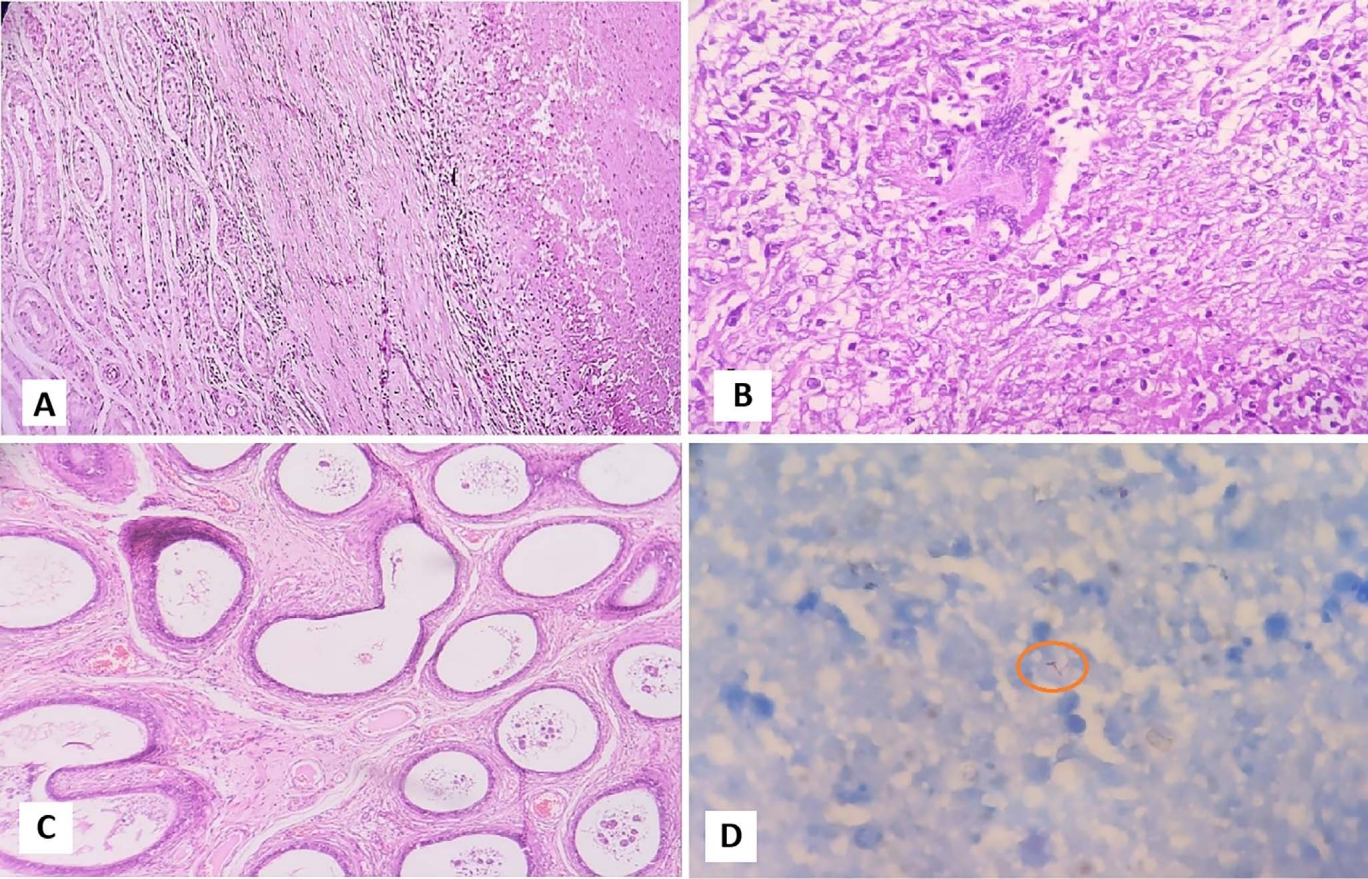
(A) Microphotograph shows unremarkable seminiferous tubules on the left side and areas of caseous necrosis surrounded by lymphocytes on the right side (hematoxylin and eosin [H&E] stain, magnification ×4). (B) Microphotograph shows high-power view of epithelioid cell granuloma and Langhans giant cells (H&E stain, magnification ×40). (C) Microphotograph shows unremarkable epididymis (H&E stain, magnification ×10). (D) Oil immersion shows acid-fast bacilli (circle) (Ziehl-Neelsen stain, magnification ×100).

Gross examination showed an enlarged testis measuring 10 × 7 × 6 cm. The cut surface showed multiple coalesced grey-white nodules involving more than half of the testicular parenchyma (Figure 1). On microscopy (Figure 2), nodules showed extensive caseous necrosis with multiple epithelioid cell granulomas and Langhans giant cells involving the testicular parenchyma. Ziehl-Neelsen stain demonstrated occasional acid-fast bacilli consistent with granulomatous orchitis of tubercular etiology. The epididymis and spermatic cord were unremarkable. No evidence of malignancy was seen. Based on these findings, isolated tuberculous testicular abscess was diagnosed.

The patient received a standard 6-month regimen of first-line antitubercular therapy: a 2-month intensive phase with isoniazid 300 mg daily, rifampicin 600 mg daily, pyrazinamide 1,500 mg daily, and ethambutol 1,200 mg daily, followed by a 4-month continuation phase with isoniazid 300 mg daily and rifampicin 600 mg daily. He tolerated therapy well, his fever and scrotal discomfort resolved, and he did not develop any pulmonary or urinary symptoms during treatment. At 12-month follow-up, the patient remained asymptomatic with a normal contralateral testis and no clinical or radiologic evidence of tuberculosis elsewhere.

## DISCUSSION

Genitourinary tuberculosis is one of the more frequent forms of extrapulmonary tuberculosis in adults, but scrotal involvement is relatively uncommon.^11^ The pathogenesis may involve hematogenous seeding from a primary pulmonary focus or retrograde spread from the prostate or seminal vesicles, although a primary testicular focus without demonstrable lesions elsewhere, as in our patient, is documented but unusual.^3,6^

The clinical presentation of tuberculous epididymo-orchitis is varied and nonspecific and frequently mimics testicular neoplasm or chronic bacterial epididymo-orchitis, making timely diagnosis challenging.^7,12^ Patients may present with painless or mildly painful scrotal swelling, often without systemic symptoms, and standard antibiotic therapy may fail, contributing to diagnostic delay. Some cases of tuberculous epididymo-orchitis have been initially managed as suspected malignancy. Orchidectomy has both diagnostic and therapeutic roles, enabling histopathologic confirmation of tuberculosis by demonstrating granulomatous inflammation with caseous necrosis and acid-fast bacilli.^6,13^

Imaging plays a critical role in the initial assessment of scrotal pathology and characterization of testicular lesions but does not show pathognomonic features specific to tuberculosis. High-resolution ultrasonography typically shows heterogeneous hypoechoic lesions in the testis or epididymis (sometimes with nodularity, calcification, or abscess formation) that may be indistinguishable from tumors.^4,7,14^ In tuberculous epididymitis, inhomogeneous enlargement and associated sinuses or wall thickening are found more frequently than in nonspecific pyogenic disease.^14^ In our patient, ultrasonography showed a lesion with characteristics of an intratesticular abscess almost completely replacing the testis without features strongly suggestive of tumor. However, the possibility of an underlying neoplasm could not be confidently excluded preoperatively. Magnetic resonance imaging may offer additional characterization but is not routinely required.

Microbiologic confirmation of genitourinary tuberculosis can be difficult because extrapulmonary disease is often paucibacillary.^15^ Acid-fast bacilli smears and the culture yield for *Mycobacterium tuberculosis* from genitourinary tuberculosis specimens have limited sensitivity, and negative results do not exclude the diagnosis.^11^ Fine needle aspiration or biopsy may help when accessible and feasible, but these procedures are not always available or advisable when malignancy cannot be ruled out.^7,16^ In such settings, orchidectomy has both diagnostic and therapeutic roles,^13^ especially in cases of a nonsalvageable testis with abscess formation, as in our patient. Histopathology demonstrating granulomatous orchitis with caseous necrosis is highly suggestive, and acid-fast bacilli presence confirms the diagnosis.^3^

Once the diagnosis is established, first-line antitubercular therapy (isoniazid, rifampicin, pyrazinamide, and ethambutol followed by a continuation phase with isoniazid and rifampicin) for 6 to 9 months is generally effective in genitourinary tuberculosis, with longer courses reserved for complicated or immunocompromised patients.^17^ Case series have demonstrated good outcomes with combined surgical and medical therapy in tuberculous epididymo-orchitis, although delayed diagnosis may predispose to infertility or other complications.^5,11,18^ In our patient, timely orchidectomy and standard antitubercular treatment resulted in complete clinical resolution, and the patient had no evidence of disseminated disease on follow-up.

This case underscores 2 important points. First, tuberculosis should remain in the differential diagnosis of testicular abscess or atypical orchitis, even in patients without pulmonary or renal disease and without obvious risk factors.^15^ Second, for patients with destructive intratesticular lesions in whom malignancy cannot be excluded and testicular salvage is unlikely, orchidectomy followed by histopathologic evaluation enables definitive diagnosis and guides appropriate antitubercular therapy.^14^

## CONCLUSION

An isolated tuberculous testicular abscess is rare and can mimic testicular malignancy or chronic infection. In regions with high tuberculosis prevalence, tuberculous testicular abscess should be considered in patients with atypical orchitis or intratesticular abscess. Orchidectomy is both diagnostic and therapeutic when testicular salvage is unlikely or malignancy cannot be excluded.

## Notes

*This article meets the Accreditation Council for Graduate Medical Education and the American Board of Medical Specialties Maintenance of Certification competencies for Patient Care and Medical Knowledge.*

## References

[1] Yuan J. Genitourinary presentation of tuberculosis. Rev Urol. 2015;17(2):102-105. 27222648 10.3909/riu0679 PMC4857903

[2] Kulchavenya E, Kim CS, Bulanova O, Zhukova I. Male genital tuberculosis: epidemiology and diagnostic. World J Urol. 2012;30(1):15-21. doi: 10.1007/s00345-011-0695-y 21604018

[3] Das A, Batabyal S, Bhattacharjee S, Sengupta A. A rare case of isolated testicular tuberculosis and review of literature. J Family Med Prim Care. 2016;5(2):468-470. doi: 10.4103/2249-4863.192334 27843865 PMC5084585

[4] Muttarak M, Peh WC, Lojanapiwat B, Chaiwun B. Tuberculous epididymitis and epididymo-orchitis: sonographic appearances. AJR Am J Roentgenol. 2001;176(6):1459-1466. doi: 10.2214/ajr.176.6.1761459 11373214

[5] Viswaroop BS, Kekre N, Gopalakrishnan G. Isolated tuberculous epididymitis: a review of forty cases. J Postgrad Med. 2005;51(2):109-111. 16006701

[6] Nakou I, Kotoulas SC, Sionidou M, Two cases of testicular tuberculosis and review of the recent literature. Int J Mycobacteriol. 2024;13(3):225-236. doi: 10.4103/ijmy.ijmy_130_24 39277883

[7] Cesilia C, Nugraha HG, Siregar S, Nataprawira HM. The challenges in diagnosing isolated epididymal tuberculosis (TB) in an adolescent male: a case report. BMC Urol. 2024;24(1):61. doi: 10.1186/s12894-024-01442-7 38504239 PMC10949577

[8] Mehboob K, Madani TA. Isolated tuberculous orchitis presented as epididymo-orchitis: an unusual presentation of tuberculosis. Urol Ann. 2022;14(2):189-195. doi: 10.4103/ua.ua_12_21 35711493 PMC9197016

[9] Lurz K, Santarelli S, Hager S, Hani AB. Pediatric intratesticular abscess managed with a testicular sparing approach: a case report. Urol Case Rep. 2021;40:101873. doi: 10.1016/j.eucr.2021.101873 34660205 PMC8502707

[10] Zaid UB, Bagga HS, Reese AC, Breyer BN. Intratesticular abscess in a solitary testicle: the case for testicle sparing management. Case Rep Med. 2013;2013:184064. doi: 10.1155/2013/184064 24198833 PMC3809365

[11] Muneer A, Macrae B, Krishnamoorthy S, Zumla A. Urogenital tuberculosis - epidemiology, pathogenesis and clinical features. Nat Rev Urol. 2019;16(10):573-598. doi: 10.1038/s41585-019-0228-9 31548730

[12] Kumar V, Swain BM, Panda S, Mohanty SS. Epididymo-orchitis with epididymal abscess in a patient with disseminated tuberculosis: a case report. J Med Ultrasound. 2025;33(3):282-284. doi: 10.4103/jmu.jmu_28_24 41018827 PMC12463358

[13] Civelli VF, Heidari A, Valdez MC, Narang VK, Johnson RH. A case of testicular granulomatous inflammation mistaken for malignancy: tuberculosis identified post orchiectomy. J Investig Med High Impact Case Rep. 2020;8:2324709620938947. doi: 10.1177/2324709620938947 PMC7493239 32618206

[14] Li S, Chen B, Fang X, A better understanding of testicular and/or epididymal tuberculosis based on clinical, ultrasonic, computed tomography, and magnetic resonance imaging features at a high-volume institute in the modern era. Quant Imaging Med Surg. 2021;11(6):2465-2476. doi: 10.21037/qims-20-1005 34079716 PMC8107328

[15] Sharma SK, Mohan A, Kohli M. Extrapulmonary tuberculosis. Expert Rev Respir Med. 2021;15(7):931-948. doi: 10.1080/17476348.2021.1927718 33966561

[16] Joshi YR, Mathur DR. Fine needle aspiration cytology – a diagnostic aid in testicular pathology. J Cytol. 2001;18(2):95-98.

[17] Wejse C. Medical treatment for urogenital tuberculosis (UGTB). GMS Infect Dis. 2018;6:Doc04. doi: 10.3205/id000039 30671335 PMC6301712

[18] Huang Y, Chen B, Cao D, Surgical management of tuberculous epididymo-orchitis: a retrospective study of 81 cases with long-term follow-up. BMC Infect Dis. 2021;21(1):1068. doi: 10.1186/s12879-021-06753-w 34654377 PMC8520285

